# The Selective Activation of p53 Target Genes Regulated by SMYD2 in BIX-01294 Induced Autophagy-Related Cell Death

**DOI:** 10.1371/journal.pone.0116782

**Published:** 2015-01-06

**Authors:** Jia-Dong Fan, Pin-Ji Lei, Jun-Yi Zheng, Xiang Wang, Shangze Li, Huan Liu, Yi-Lei He, Zhao-Ning Wang, Gang Wei, Xiaodong Zhang, Lian-Yun Li, Min Wu

**Affiliations:** 1 Department of Biochemistry and Molecular Biology, College of Life Sciences, Wuhan University, Wuhan, Hubei 430072, China; 2 Department of Cell Biology and Development Biology, College of Life Sciences, Wuhan University, Wuhan, Hubei 430072, China; 3 CAS-MPG Partner Institute for Computational Biology, Shanghai Institutes for Biological Sciences, Chinese Academy of Sciences, Shanghai 200031, China; National Cancer Center Research Institute, JAPAN

## Abstract

Transcription regulation emerged to be one of the key mechanisms in regulating autophagy. Inhibitors of H3K9 methylation activates the expression of LC3B, as well as other autophagy-related genes, and promotes autophagy process. However, the detailed mechanisms of autophagy regulated by nuclear factors remain elusive. In this study, we performed a drug screen of SMYD2^-/-^ cells and discovered that SMYD2 deficiency enhanced the cell death induced by BIX01294, an inhibitor of histone H3K9 methylation. BIX-01294 induces accumulation of LC3 II and autophagy-related cell death, but not caspase-dependent apoptosis. We profiled the global gene expression pattern after treatment with BIX-01294, in comparison with rapamycin. BIX-01294 selectively activates the downstream genes of p53 signaling, such as p21 and DOR, but not PUMA, a typical p53 target gene inducing apoptosis. BIX-01294 also induces other autophagy-related genes, such as ATG4A and ATG9A. SMYD2 is a methyltransferase for p53 and regulates its transcription activity. Its deficiency enhances the BIX-01294-induced autophagy-related cell death through transcriptionally promoting the expression of p53 target genes. Taken together, our data suggest BIX-01294 induces autophagy-related cell death and selectively activates p53 target genes, which is repressed by SMYD2 methyltransferase.

## Introduction

Protein methylation on histones is initially well demonstrated in transcription regulation and chromatin structure [1, 2]. Later, methylation on non-histone proteins is also proved to be one of the key steps in regulating protein functions [3]. The protein methyltransferase family of SET and MYND domain containing proteins is of important functions in tumorigenesis and development processes [4]. These proteins contain an atypical SET domain, which is split into two parts by one MYND domain [4]. SMYD proteins exert their function by methylating proteins on lysines, among which SMYD2 (SET and MYND domain containing 2) is the mostly studied.

SMYD2 is initially identified as a methyltransferase for histone H3K36 and H3K4 [5, 6]. Till now, the SMYD2 target sites on chromatin are still not well demonstrated, however, since it mainly localizes in the cytoplasma, SMYD2 has important functions on non-histone proteins. Multiple proteins were identified as the substrates of SMYD2, such as p53 (tumor protein p53), Rb (retinoblastoma 1), HSP90 (heat shock protein 90kDa), PARP1 (poly (ADP-ribose) polymerase 1) and ESR1 (estrogen receptor 1) [7–11]. SMYD2 methylates p53 at Lys370 and represses p53 transcription activity [7]. Since p53 and Rb are among the most well-known tumor suppressor genes, SMYD2 is considered a potential oncogene. Several studies reported that SMYD2 is overexpressed in the tumor cells lines and patients’ tissues of some cancer types, including esophageal squamous cell carcinoma and acute lymphoblastic leukemia, which suggests SMYD2 as a potential drug target in these cancers [9, 12, 13].

The tissues with most abundant SMYD2 expression include heart, brain and muscle [14]. Surprising, SMYD2 deficiency in cardiomyocyte is dispensable for heart development [14]. Recently, one report proved SMYD2 represses p53 activity and cardiomyocyte apoptosis induced by cobalt chloride, which suggested SMYD2 as a regulatory protein in stress response [15].

In order to explore SMYD2’s novel physiological functions in other pathways, we carried out a functional drug screen in SMYD2 knockout cell line. We identified SMYD2 deficiency enhanced cell death induced by BIX-01294. BIX-01294 is the first inhibitor identified against histone H3K9 methyltransferase G9a and strongly impairs global histone H3K9 di- and trimethylation [16]. It is able to regulate differentiation and block cancer cell growth [17–20]. Recently, BIX-01294 was reported to be an autophagy inducer in multiple cell lines [21]. EHMT2/G9a (euchromatic histone-lysine N-methyltransferase 2) and H3K9 methylation were also shown to be involved in autophagy via mediating the transcription of key autophagy genes, such as LC3B [22, 23]. Autophagy is an important cellular process to recycle unwanted organelles, metabolic energy and metabolites in the time of starvation or other stress conditions [24–26]. Different from the classical pathway induced by starvation, a new mechanism driven by transcriptional factors in the nuclear, such as inhibition of histone H3K9 methylation, emerged to be critical in inducing autophagy [22]. However, the detailed mechanisms of autophagy induced by inhibition of H3K9 methylation remain elusive.

In this study, we further investigated the mechanisms of BIX-01294-induced autophagy by high throughput sequencing and found that SMYD2 regulates autophagy related cell death induced by BIX-01294, which is dependent on p53 and the transcription of its target genes.

## Materials and Methods

### Cell lines and reagents

U2OS cell line was purchased from Cell Bank of Chinese Academy of Science. HCT116 and U2OS cells were grown in DMEM (Invitrogen) supplemented with 10% fetal bovine serum (Hyclone) and 1x penicillin/streptomycin (HyClone) at 37°C with 5% CO_2_. Antibodies against LC3 II (Sigma), β-actin (Abclonal), p53 (CST) and caspase3 (CST) were purchased from indicated merchandiser. Rabbit anti-SMYD2 was raised in the lab.

### Generation of knockout cell line

The genetic SMYD2 knockout cell line derived from colorectal cancer cells HCT116 was generated as previously described [27, 28]. Briefly, the genomic DNA of HCT116 was extracted and the homologous arms of the target site were amplified and cloned into the pAAV-LoxP-Neo targeting vector. HEK 293 cells (1×10^5^) were transfected with the targeting construct and packaging vectors (pAAV-RC and pHelper) to generate recombinant adeno-associated viruses (rAAVs). Three days later, the cells were harvested and underwent freeze-thaw in liquid nitrogen three times. The supernatant was harvested as the rAAV stock. HCT 116 cells (1×10^5^) were infected with rAAV virus for 48h and were then split into a series of 96-well plates using limited dilution methods to obtain single clones. After 2 weeks of selection in G418-containing medium, the clones were screened by genomic PCR and positive clones were grown up. Then, the clones were infected with GFP-Cre Adenovirus to cut the resistant Neo gene. The infected cells were screened by PCR to screen for the correct heterozygous clones. Subsequently, the second round of gene targeting was performed as above to get the homozygous clones.

### Cell viability assay

Cell viability was performed by the MTT assay as previously described [29]. Briefly, cells were split at 5×10^3^ per well in 96-well plates. After 24 h of culture, cells were treated with varying concentrations of drugs for 48 or 72h. Following incubation of cells in each well with MTT (0.25μg) for 4h at 37°C, the medium with the formazan sediment was dissolved in 50% DMF and 30% SDS (pH4.7). The absorption was read at 570nm.

### Immunofluorescent staining

Cells were cultured on the cover slips and fixed with freezing methanol after washed twice in PBS. The cover slips were then washed three times by PBS and blocked in PBS with 1% BSA for 10min. The cover slips were hybridized with first and second antibodies for one hour, respectively. Then the slips were mounted with prolong anti-fade kit (Invitrogen) and observed with fluorescent microscopy.

### Cell cycle analysis with flow cytometry

After treated with BIX-01294 for 24h, cells were harvested after digestion with 0.05% Trypsin-EDTA. The cells were then washed twice with PBS and fixed in ice-cold 70% ethanol overnight. Fixed cells were washed twice with PBS and stained in PBS containing propidium iodide (PI, 50μg/mL) and RNase (100μg/mL) for 30min at 37°C. Cell cycle analysis was performed on an Epics XL-MCL flow cytometer (Beckman Coulter) with System II (version 3.0) software (Beckman Coulter). Additional analysis of cell cycle distribution was determined using Flowjo software.

### Reverse transcription and quantitative PCR

Cells were scraped down and collected by centrifugation. Total RNA was extracted with RNA extraction kit (Yuanpinghao) according to manufacturer’s manual. Approximately 1μg of total RNA was used for reverse transcription with a first strand cDNA synthesis kit (Toyobo). The amount of mRNA was assayed by quantitative PCR. β-actin was used to normalize the amount of each sample. Assays were repeated at least three times. Data shown were average values ± SD of one representative experiment. All primer and siRNA sequences are presented in Tables H and I in S1 File.

### Profiling of global gene expression

mRNA-seq library was performed by using Illumina TruSeq library construction kit. Using 5μg total RNA as initiation, and then prepared according to the manufacturer’s instruction. mRNA-seq libraries were sequenced using HiSeq2000 for 100bp paired-end sequencing. Quality control of mRNA-seq data was performed using Fatsqc, and then, low quality bases were trimmed. After quality control, data were mapped to hg19 genome reference by Tophat2 and allow maximum 2 mismatch [30]. Cufflinks was used to find out differential expression genes. Gene ontology analysis was performed using DAVID (http://david.abcc.ncifcrf.gov). The data have been uploaded to GEO database and can be found at the following URL: http://www.ncbi.nlm.nih.gov/geo/query/acc.cgi?token=ytkbseoivhcrpab&acc=GSE61255.

## Results

### Generation of SMYD2 deficient cell from HCT116 cell line

**Figure 1:**
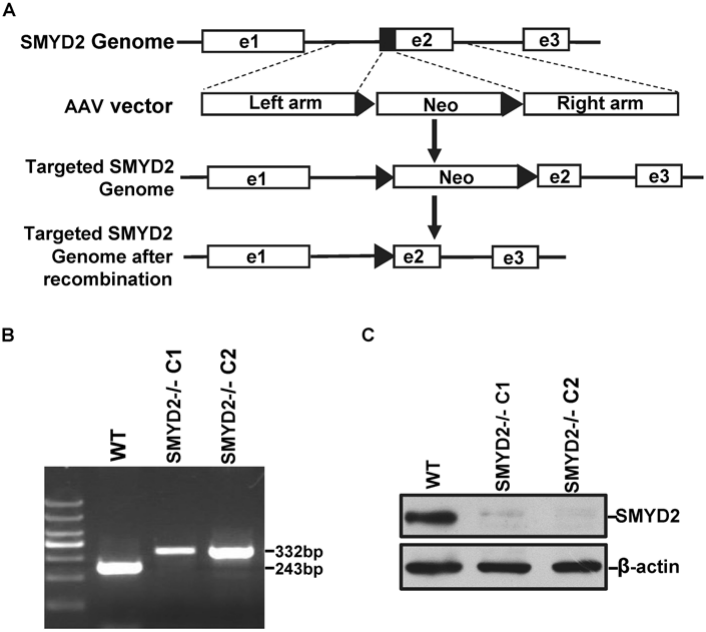
Generation of SMYD2 deficient cell in HCT116 cell line. **(A)** A sketch map of the strategy to generate SMYD2^-/-^ cell line by homologous recombination. **(B)** Genomic PCR was performed to identify SMYD2^+/+^ (243bp) and SMYD2^-/-^ cell lines (332bp) respectively. **(C)** Whole cell lysates of SMYD2^+/+^ and SMYD2^-/-^ cells were analyzed by immunoblotting with anti-SMYD2 and anti-β-actin antibodies.

To study the functions of SMYD2, we generated SMYD2 knockout cell lines by homologous recombination in HCT116 cell line. The design is shown in Fig. 1A. Exon 2 in SMYD2 genes was partially deleted, which generated a shift of the open reading frame in the transcribed mRNA. Two successful clones were finally identified. The successful recombination in the both alleles of target gene were confirmed by genomic PCR (Fig. 1B), and the protein expression was confirmed by western blot (Fig. 1C).

### Drug screen in SMYD2 deficient cells

**Figure 2:**
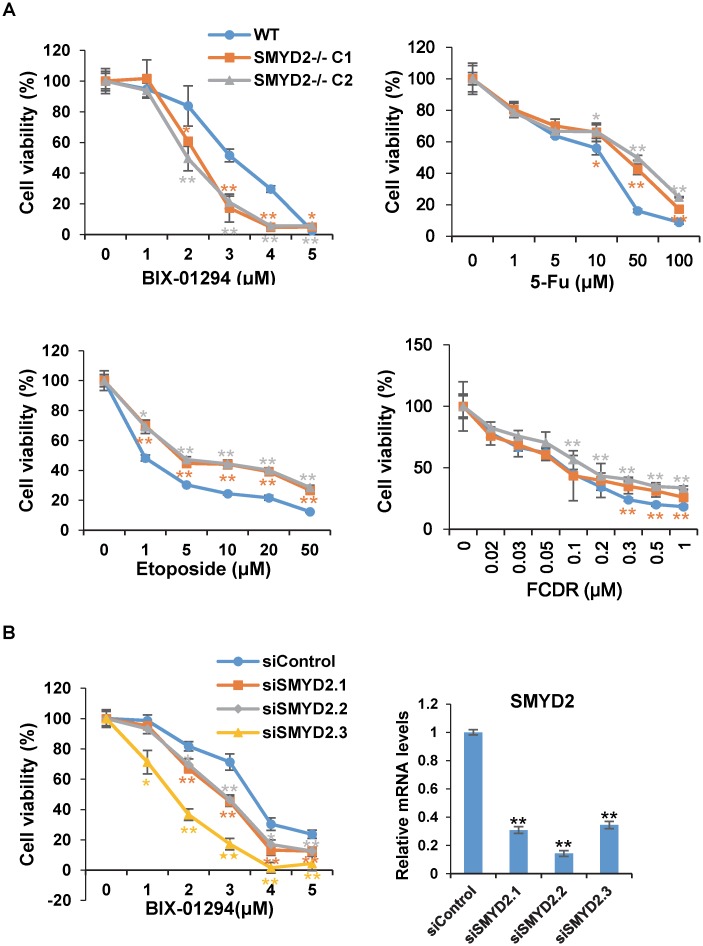
Drug screening of SMYD2^+/+^ and SMYD2^-/-^ cells. **(A)** SMYD2^+/+^ and SMYD2^-/-^ cells were treated with various concentrations of BIX-01294, 5-Fu, Etoposide, FCDR for 72h. Cell viability was measured by MTT assay. **(B)** HCT 116 cells were transfected with SMYD2 siRNAs for 48h, followed by BIX-01294 treatment for 24h. Cell viability was measured by MTT assay. The knockdown efficiency was measured by quantitative RT-PCR. Data are represented as mean ± SEM. P-values were calculated using Student’s t-test (*P < 0.05; ** P < 0.01).

A drug screen was carried out with SMYD2^+/+^ and SMYD2^-/-^ cell lines. MTT assay was used to determine the cell viability after drug treatment as described in Materials and Methods. The drugs used in the screen and their related functions are shown in Table A in S1 File. Part of the screen results are shown in Fig. 2A. SMYD2 deficiency increases cell viability with 5-Fu and etoposide, while decreases cell viability with BIX-01294 (Fig. 2A). To further confirm our results, we knocked down SMYD2 in the parent HCT116 cells and treated them with BIX-01294 (Fig. 2B). The cells with SMYD2 knockdown is also more sensitive to BIX-01294 (Fig. 2B), which suggests our discovery in the drug screen was unlikely an artificial effect. 5-Fu and etoposide are classical chemotherapy drugs inducing DNA damage response pathway. Previous studies reported that SMYD2 knockdown enhances cell cycle arrest and apoptosis induced by DNA damage reagents. This is conflict with our results. After careful experiments, we found that SMYD2 knockdown could not repeat the results in SMYD2^-/-^ cell (data not shown). We then concluded that it does not reflect the real nature of SMYD2 in the cell. Then we focused on the roles of SMYD2 in BIX-01294 induced cell death.

### Induction of autophagy in HCT116 by BIX-01294

BIX-01294 is a strong inhibitor of histone H3K9 methylation. It is also a potent inducer of cell death at the concentration of 2–5μM (Fig. 2A&B). We firstly tried to determine whether BIX-01294 induces apoptosis in the cell. The cleaved CASP3 and PARP1 are hall markers for apoptosis. However, we could not detect any cleaved forms of the above proteins in multiple experiments. We also failed to detect apoptotic cells after BIX-01294 treatment with a commercial Annexing V kit with flow cytometry. These suggested that the cell death triggered by BIX-01294 is not caspase-dependent apoptosis.

**Figure 3:**
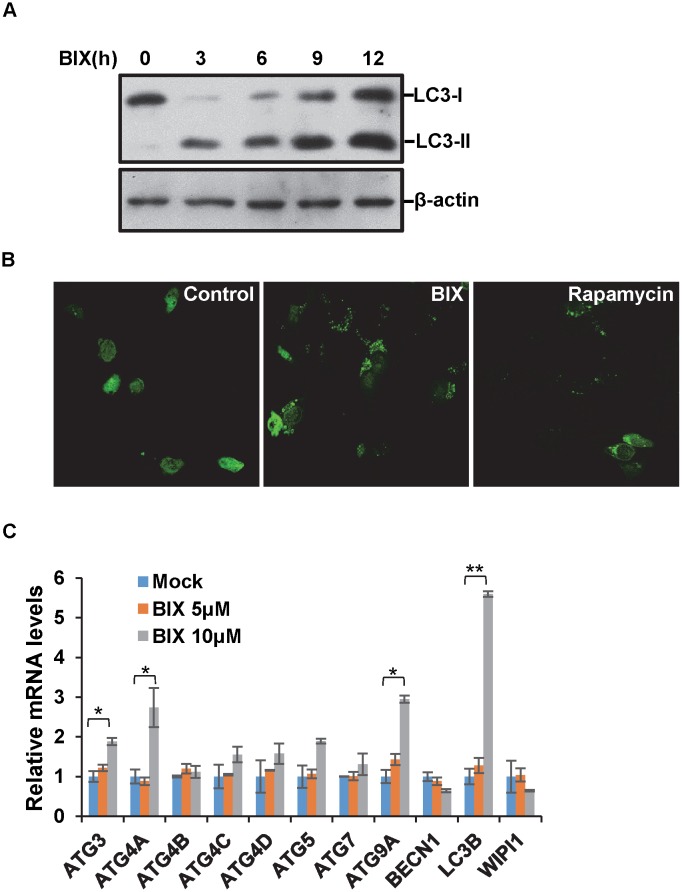
BIX-01294 induces autophagy in HCT 116 cells. **(A)** HCT116 cells were treated with 5μM BIX-01294 for the indicated time. Western blotting was performed using antibodies of LC3B and β-actin. **(B)** HCT116 were transfected with GFP-LC3, followed by 5μM BIX-01294 or 1μM rapamycin for 12h. Cells were examined with fluorescence microscopy. **(C)** HCT116 cells were treated with 5μM or 10μM BIX-01294 for 12h, the mRNA levels of genes involved in autophagy process were measured by quantitative RT-PCR.

Then we thought if BIX-01294 affect other events in determining cell fate. We analyzed the cell lysates with western bolt by using anti-LC3 antibody, a hall marker for autophagy. The results showed that LC3 II is formed after BIX-01294 treatment, which suggests autophagy was induced in the cell (Fig. 3A). The confocal images also showed that after transfection of GFP-LC3 and BIX-01294 treatment, the condensed LC3 II spots were observed in the cytoplasm compared the diffused distribution in the control sample, which is another hall marker for autophagy (Fig. 3B). Our data suggest that BIX-01294 induces autophagy in multiple cell lines, as well as non-apoptotic cell death. When we were preparing the manuscript, one group reported that BIX-01294 induces autophagy in multiple cell lines [21]. Our data perfectly matched theirs and came to the same conclusion.

Since BIX-01294 is an inhibitor to histone H3K9 methylation, we wonder if it induces autophagy by transcription regulation. We studied the mRNA level of several key genes in autophagy pathway. After BIX-01294 treatment, the expression of LC3B, ATG9A and ATG4A increased, but not ATG4B/C, ATG7, BECN1 and WIPI1 (Fig. 3C). These suggested the alteration of gene expression pattern induced by BIX-01294 plays important roles in autophagy. A recent study reported that inhibition of histone H3K9 methyltransferase G9a activates the expression of LC3B [23]. Our data confirmed their discovery and identified more autophagy-related genes involved in the process.

### SMYD2 does not regulate BIX-01294-induced autophagy

**Figure 4:**
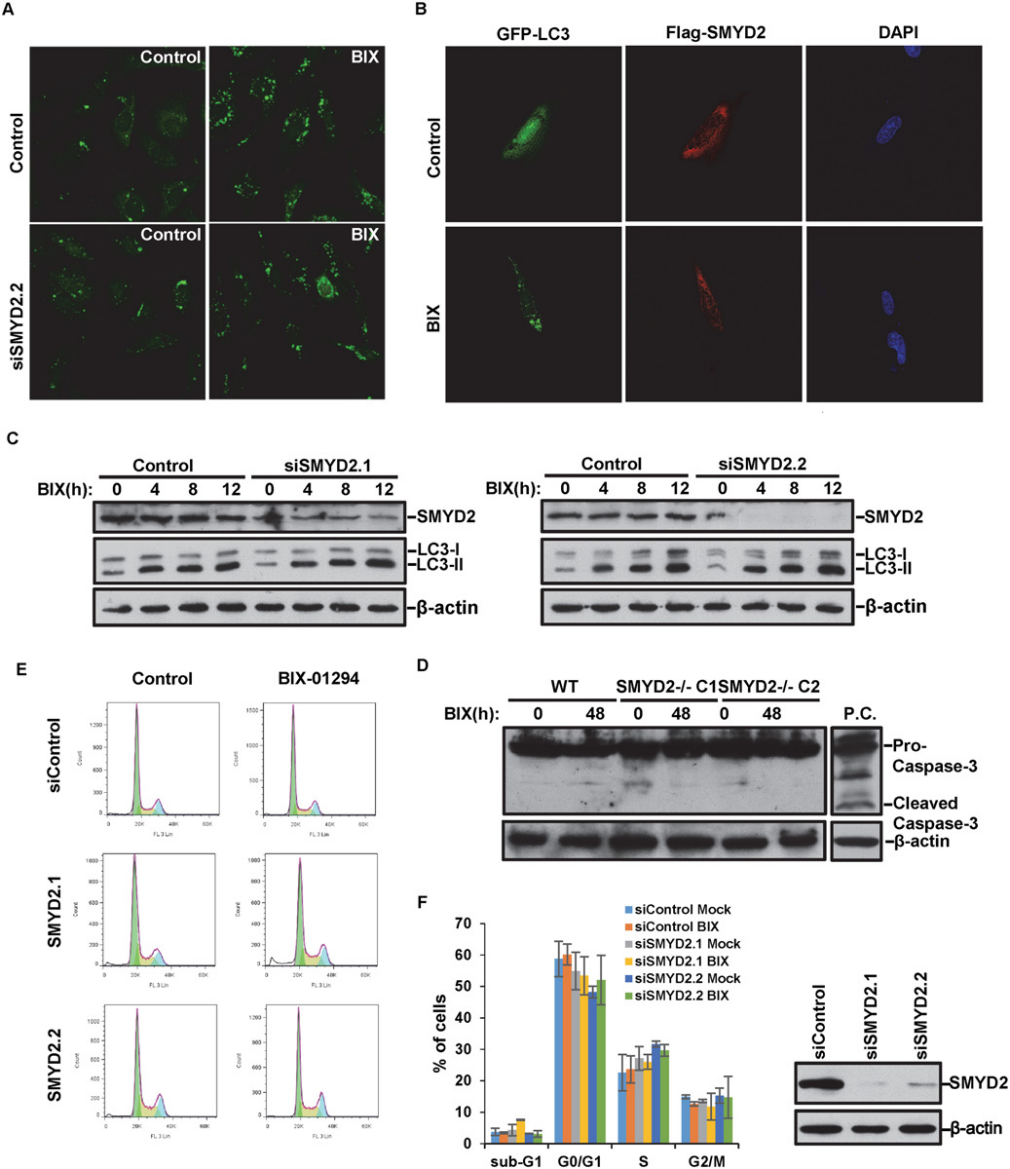
SMYD2 deficiency does not impair BIX-01294-induced autophagy process. **(A)** Cells were transfected with SMYD2 siRNA for 48h, followed by 5μM BIX-01294 for 4h. Cells were immunostained with anti-LC3 antibody for immunofluorescence analysis. **(B)** Cells were transfected with Flag-SMYD2 and GFP-LC3 plasmids. 24h later, cells were treated with 5μM BIX-01294 for 2h and then collected for fluorescence microscopy. **(C)** HCT116 cells were transfected with indicated SMYD2 siRNAs for 48h, followed by treatment with 5μM BIX-01294 for indicated times. Western blot analysis was performed using anti-SMYD2, anti-LC3, and anti-β-actin antibodies. Results shown are representatives of at least three independent experiments. **(D)** Whole cell lysates of SMYD2^+/+^ and SMYD2^-/-^ cells treated with 5μM BIX-01294 for 48h were analyzed by western blotting with anti-caspase3 and anti-β-actin antibodies. **(E)** HCT116 cells were transfected with SMYD2 siRNAs for 48h, and then treated with 5μM BIX-01294 for 24h. Cell cycle distribution was analyzed with flow cytometry. **(F)** The histogram (Left) and the western blotting of SMYD2 (right) of the cell cycle analysis in (E).

After determining that BIX-01294 induces autophagy in the cell, we asked whether SMYD2 regulates the autophagy process. Surprisingly, though SMYD2 deficiency enhances BIX-01294-induced cell death, it does not affect the formation of LC3 II (Fig. 4C). To further confirm the discovery, we observed the formation of LC3 II under microscope. When comparing wild type and SMYD2 deficient cells, we did not see obvious difference of either the percentage or the average numbers of cells with condensed LC3 II staining (Fig. 4A). The data suggested SMYD2 deficiency does not affect the autophagy process. Our data also showed that exogenous expressed SMYD2 does not co-localize with autophagic LC3 II (Fig. 4B). Moreover, SMYD2 knockout does not lead to caspase 3 cleavage under BIX-01294 treatment, which indicated that the cell death enhanced by SMYD2 deficiency is not apoptosis either (Fig. 4D). SMYD2 deficiency also does not significantly affect cell cycle with BIX-01294 treatment (Fig. 4E&F).

### BIX-01294 induces autophagy by different mechanisms compared with rapamycin

**Figure 5:**
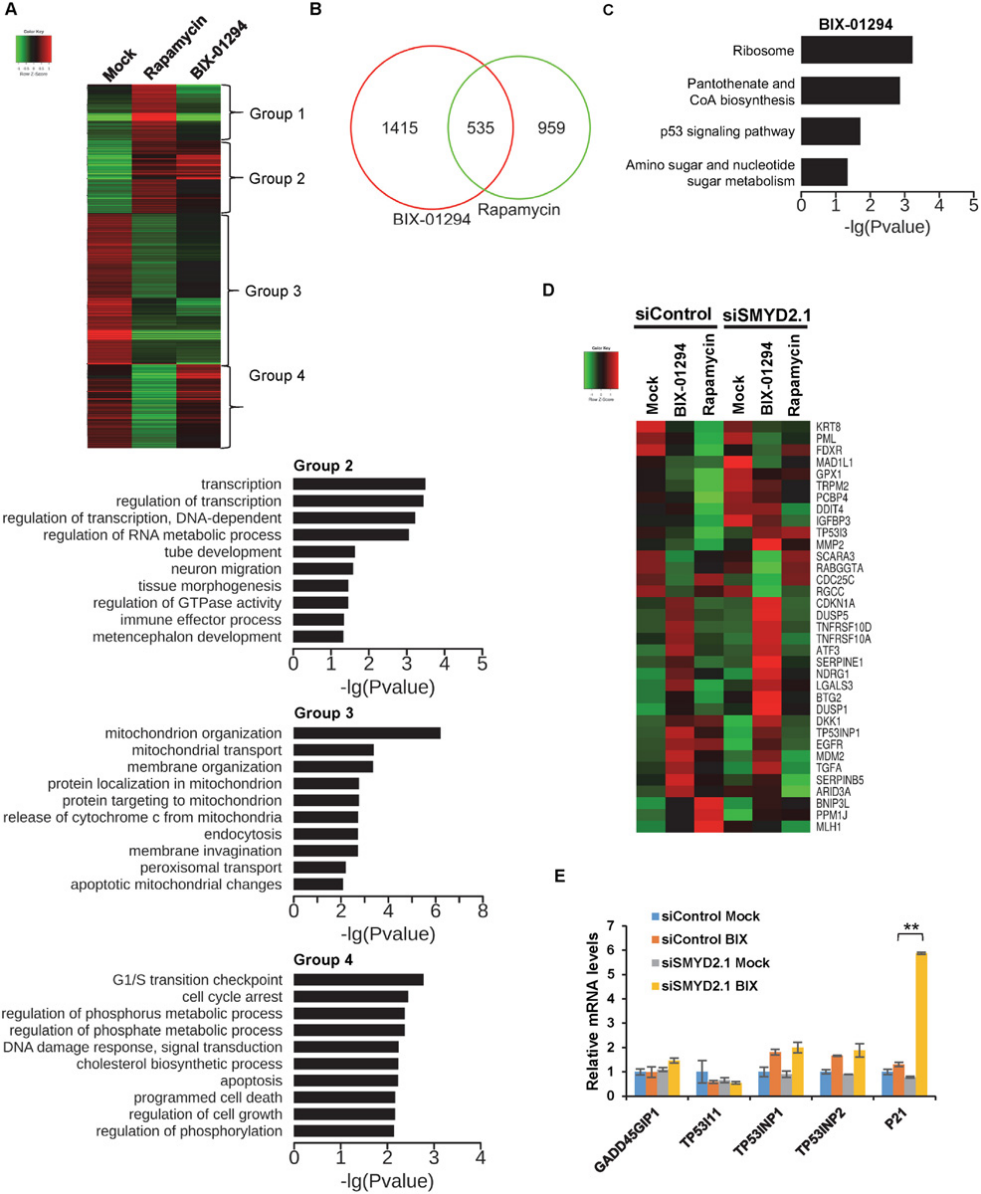
Genome wide gene expression profiles of the cells treated with BIX-01294 and rapamycin. HCT116 cells were transfected with control or SMYD2 siRNA, and treated with H_2_O, BIX-01294 and rapamycin respectively. **(A)** Clustering of the different expressed genes induced by rapamycin among the three samples showed that they can be divided into four groups based on their expression patterns. Each group was analyzed by GO analysis except group 1, which no significant biological process was found. **(B)** Venn diagram shows the numbers of gens regulated by BIX-01294, rapamycin alone or both. **(C)** GO analysis of signaling pathways enriched by different expressed genes uniquely activated by BIX-01294. **(D)** SMYD2^+/+^ and SMYD2^-/-^ cells were treated with H_2_O, BIX-01294 and rapamycin respectively. Different expressed p53 target genes were shown in the heat map. **(E)** The mRNA level of the typical p53 target genes was examined by quantitative PCR in the indicated samples.

Our data suggested that BIX-01294 induce autophagy through transcription regulation (Fig. 3C). To further investigate the mechanisms of BIX-01294-induced autophagy, we compared the global gene expression profiles of BIX-01294 and rapamycin treatment, using high throughput mRNA sequencing. Before sample submission, we analyzed the cells by western blot to ensure the similar amount of LC3 II was induced by the above two drugs. BIX-01294 treatment changed the expression of 1950 genes (more than 1.5 folds), and 1494 genes for rapamycin (Fig. 5A and Tables B-C in S1 File). Among them, 535 different expressed genes (DEG) were overlapped (Fig. 5B). We took the DEGs between rapamycin and control, cluster them among all three samples and grouped into 4 groups (Fig. 5A). The genes in group 1 are activated by rapamycin but not BIX-01294 (Fig. 5A). We failed to link them with any biological process with GO analysis. The genes in group 2 are both activated by the two drugs and are mainly related with transcriptional regulation and RNA metabolism (Fig. 5A). The genes in group 3 are down regulated by both drugs and they are mostly related with processes involving mitochondria (Fig. 5A). Those in group 4 are down regulated by rapamycin, but did not change too much with BIX-01294. They are mostly related with cell cycle, metabolism and cell death (Fig. 5A).

We also analyzed to see if the DEGs reflect the change of signaling pathways. We took the DEG specifically induced by BIX-01294 and categorized them into pathways related with ribosome, metabolisms, and p53 signaling (Fig. 5B). No signaling pathways were significantly enriched by rapamycin-induced DEGs. These suggested that the alterations of gene expression pattern induced by the two drugs are of great difference. We also found that ATG4A and ATG9A, two autophagy-related genes we identified previously, increases their mRNA expression after BIX-01294 treatment, but not rapamycin (Tables B-C in S1 File).

### BIX-01294 selectively induces the expression of p53 downstream genes

By studying the global gene expression profiling, we surprisingly found that p53 target genes are enriched in the DEG induced by BIX-01294, including CDKN1A (cyclin-dependent kinase inhibitor 1A, p21), MDM2 (transformed mouse 3T3 cell double minute 2), PMAIP1(phorbol-12-myristate-13-acetate-induced protein 1, NOXA), TP53I11 (tumor protein p53 inducible protein 11, PIG11), TP53INP1 (tumor protein p53 inducible nuclear protein 1), TP53INP2 (tumor protein p53 inducible nuclear protein 2, DOR), GADD45GIP1 (growth arrest and DNA-damage-inducible, gamma interacting protein 1) (Fig. 5B and Table B in S1 File). None of the above genes appeared in the DEG list of rapamycin (Table C in S1 File), suggesting the activation of p53 downstream genes is BIX-01294 specific. We confirmed the results by quantitative RT-PCR (Fig. 5E). Interestingly, we noticed that the change of one of the above genes, TP53I11, is opposite to others. Its mRNA level was repressed rather than elevated. Moreover, BIX-01294 did not active the expression of PUMA, the typical apoptosis-related genes, and DRAM1, a gene involved in both autophagy and apoptosis (Table B in S1 File). PUMA, DRAM1 and many other genes are well defined p53 target genes, which are activated during many stress conditions, such as DNA damage [31]. For example, they are activated by p53 following treatment of DNA damage drug, 5-fluorouracil (Table D in S1 File). As comparison, the typical p53 target gene regulating autophagy, TP53INP2/DOR, is activated by BIX-01294 but not 5-fluorouracil. Together, our data suggest BIX-01294 treatment selectively activates the expression of p53 target genes related with cell cycle and autophagy, but not apoptosis, which contributes to the autophagy process.

### The gene expression profiles regulated by SMYD2 under BIX-01294 treatment

SMYD2 is a methyltransferase of p53 and regulates its transcription activity. It is expected that SMYD2 deficiency enhances p53 target genes’ expression under BIX-01294 treatment. We indeed observed that p53 target genes induced by BIX-01294 further elevated their expression with SMYD2 knockdown (Fig. 5D & Tables B-F in S1 File). Moreover, some of the target genes which is not activated in control cells, is activated in siSMYD2.2 cells (Fig. 5D). For those down regulated by BIX-01294, SMYD2 deficiency does not elevate their expression. Since rapamycin does not activate p53 signaling, SMYD2 deficiency does not obviously alter the expression of p53 target genes (Fig. 5D and Table G in S1 File). These suggest that p53 signaling is critical in the BIX-01294-induced cell death enhanced by SMYD2 deficiency.

**Figure 6:**
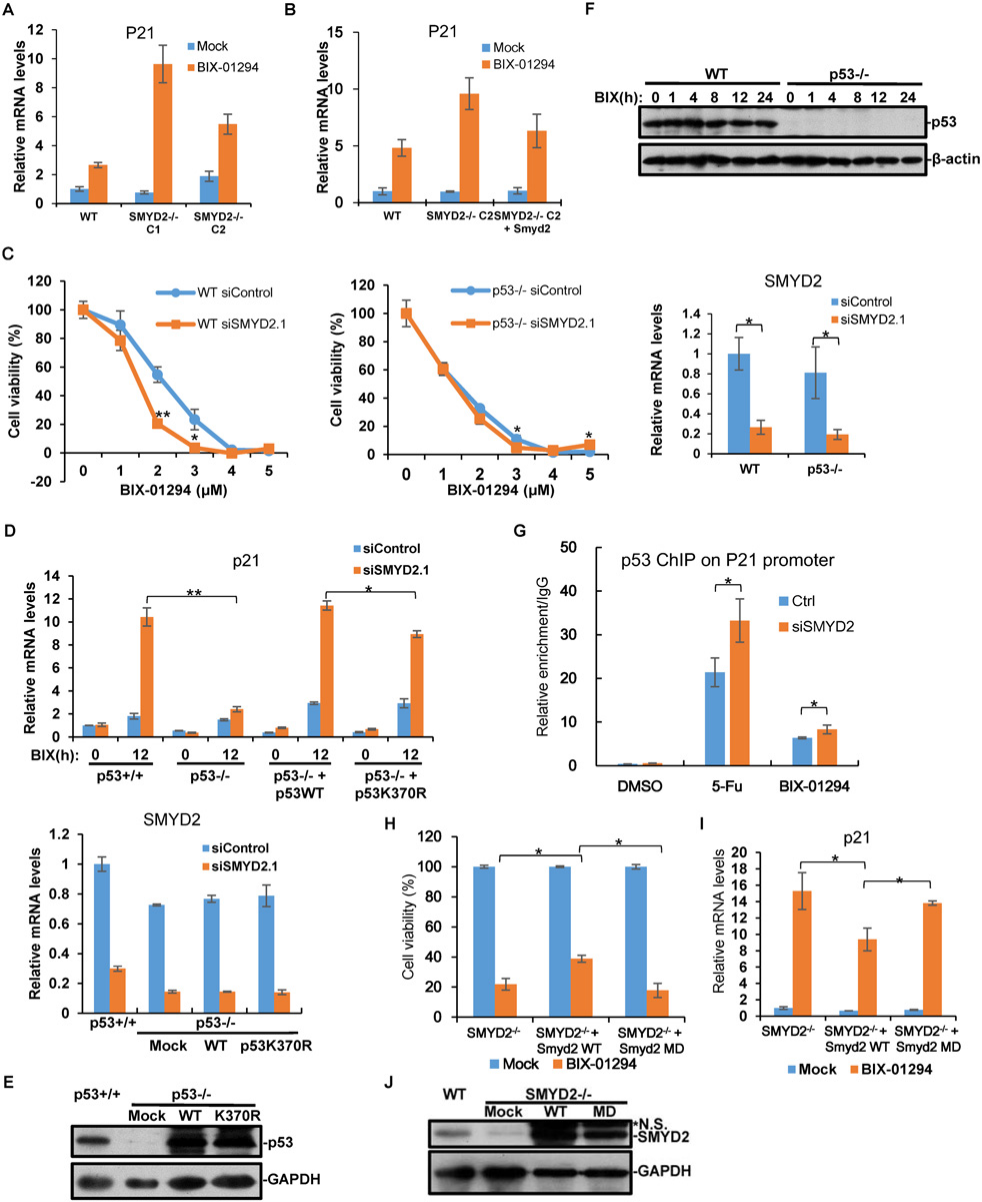
SMYD2 deficiency promotes BIX-01294-mediated cell death through p53 pathway. **(A)** The p21 mRNA levels in SMYD2^+/+^ and SMYD2^-/-^ cells with 5μM BIX-01294 were examined by quantitative RT-PCR. **(B)** Flag-tagged SMYD2 was exogenous expressed in SMYD2^-/-^ cell. SMYD2^-/-^ and Flag-SMYD2 cells were treated with 5μM BIX-01294 for 12h and the mRNA levels of p21 were determined by quantitative RT-PCR. **(C)** p53^+/+^ and p53^-/-^ HCT 116 cells were transfected with SMYD2 siRNA for 48h, followed by BIX-01294 treatment. Cell viability was analyzed by MTT assay. The knockdown efficiency was measured by quantitative RT-PCR. **(D)** The indicated four cell lines were transfected with SMYD2 siRNA for 48h, and treated with 5μM BIX-01294 for 12h. The mRNA levels of p21 were determined by quantitative RT-PCR. **(E)** p53 wild type and K370R mutant were transfected into p53^-/-^ cell line and stable expressed cell lines were screened. p53 expression in the cell lines used in (D) were examined by western and GAPDH as loading control. **(F)** Whole cell lysates of WT and p53^-/-^ HCT 116 cells upon 5μM BIX-01294 treatment for indicated times were analyzed by western blotting with anti-p53 and anti-β-actin antibodies. **(G)** HCT116 cells were transfected with SMYD2 siRNA and treated with indicated drugs. The bound p53 was measured with ChIP assay on the p53 response element of p21 promoter. **(H)** The indicated cells were treated with 5μM BIX-01294 and cell viability was measured with MTT assay. (I) p21 mRNA level was examined by quantitative RT-PCR for samples in (H). **(J)** A mouse version of Smyd2 wild type cDNA and catalytic dead mutant SMYD2MD were stably expressed in SMYD2^-/-^ cell lines respectively. The expression of Smyd2 was measured by western and GAPDH as loading control. P-values were calculated using Student’s t-test (*P < 0.05; ** P < 0.01).

We confirmed the sequencing results by quantitative RT-PCR (Fig. 5E). To further ensure the elevated expression of p53 target genes is due to the loss of SMYD2, we reintroduced SMYD2 back into SMYD2^-/-^ cell lines and examined p21 expression after BIX-01294 treatment (Fig. 6A&B). The re-expression of SMYD2 suppressed p21 mRNA level as that in wild type cells, which further proved that SMYD2 regulates its expression during BIX-01294 treatment.

### The autophagy related cell death enhanced by SMYD2 deficiency is dependent on p53

The above data suggest p53 signaling is critical in BIX-01294-induced cellular process. To further prove it, we utilized the p53 knockout cell lines derived from HCT116. We knocked down SMYD2 in p53^+/+^ and p53^-/-^ cells respectively, and then measured the cell viability. SMYD2 deficiency in p53^-/-^ cells had the same survival rate under BIX-01294 treatment, compared with the control samples (Fig. 6C), which proved p53 is the key molecule essential for SMYD2 mediated cell death with BIX-01294 treatment. We further measured the p21 mRNA level in the above cell line. The expression of p21 was greatly enhanced with SMYD2 knockdown in p53^+/+^ cells; however, it was not activated in p53^-/-^ cells (Fig. 6D & E). We did not observe significant difference of LC3 II formation comparing p53^+/+^ and p53^-/-^ cell lines (data not shown), which suggests other mechanisms governs the formation of LC3 II induced by BIX-01294. All these suggest that p53 signaling is critical in BIX-01294 induced cell death process.

The main regulatory mechanism of p53 signaling is via regulation of p53 protein stability. We studied if p53 protein level is stabilized after BIX-01294 treatment. Surprisingly, p53 protein kept at the same level with BIX-01294 treatment (Fig. 6F), which suggests the activation of p53 signaling during BIX-01294 treatment is mainly through regulation of p53 transcriptional activity, but not protein stability.

SMYD2 is a methyltransferase for p53 at lysine 370 [7]. To further study if SMYD2-mediated p53 methylation is involved in the autophagy related cell death induced by BIX-01294, we generated cell lines stably expressing p53 wild type or K370R mutant in p53^-/-^ cell (Fig. 6E). Under the condition of SMYD2 deficiency, p53 expression restored p21 activation by BIX-01294 in p53^-/-^ cell; however, p53 K370R mutant only partially restore p21 expression (Fig. 6D). These suggest p53 K370 methylation plays a role in BIX-01294-induced gene expression. The bound p53 on the p21 promoter also examined by ChIP assay. SMYD2 deficiency enhanced the p53 amount on p21 promoter (Fig. 6G), although the total p53 protein did not change (Fig. 6F). To further confirm its methyltransferase activity is critical for SMYD2-mediated autophagy related cell death, we stably expressed mouse Smyd2 wild type cDNA or catalytically dead mutant Smyd2MD (H207A) [7] into SMYD2^-/-^ cell (Fig. 6J). The restore of Smyd2 gene increased cell viability almost two folds, but Smyd2MD did not (Fig. 6H). Smyd2 restoration also repressed p21 expression activated by BIX-01294, but not Smyd2MD (Fig. 6I). Taken together, our data indicated that p53 methylation by SMYD2 regulates autophagy related cell death induced by BIX-01294.

## Discussion

Many drugs and conditions induce autophagy process. The molecular pathway of autophagy triggered by starvation has been well defined; however, that triggered by other factors remain elusive. Recently, BIX-01294, an inhibitor of histone H3K9 methyltransferase G9a, was reported to be a potent autophagy-inducible drug. It eventually induces non-apoptotic cell death but the mechanism is not clear. Our studies nicely confirmed their discoveries. Furthermore, by utilizing next generation sequencing, we found that BIX-01294 altered the transcription of genes involved in ribosome, metabolisms and p53 signaling, as well as several autophagy-related genes, including LC3B, ATG4A, ATG9A, TP53INP2/DOR and TP53INP1. In comparison, rapamycin, a classical autophagy inducer does not regulate the transcription of known genes directly involved in autophagy. Both rapamycin and BIX-01294 enhance the expression of genes involved in transcription regulation and repress genes related with mitochondria. These suggested that the two drugs also share similar mechanisms in regulating autophagy and the regulation of transcription and mitochondria function are critical in the autophagy process. Since BIX-01294 is a small molecule inhibiting histone H3K9 methylation, which is well-known markers repressing transcription, it is highly possible that BIX-01294 induces autophagy mainly through transcriptional regulation. A previous study has reported that LC3 transcription is activated upon repressing H3K9 methylation. We did observe the same phenomenon and we revealed more autophagy-related genes induced by the drug. In the previous studies, the regulation of autophagy by transcription did not draw enough attention in the field. Our study suggested the transcriptional regulation could be also important.

By performing a drug screen, we found that SMYD2 deficiency sensitizes the cells to BIX-01294 induced cell death. We further proved that p53 signaling is critical in the process. Unlike rapamycin, BIX-01294 activates the expression of p53 target genes and SMYD2 represses p53 transcription activity. SMYD2 deficiency increases autophagy-related cell death via up regulates the expression of p53 target genes. Thus SMYD2 plays a role in regulating autophagy-related cell death through repression of p53 signaling. However, the roles of p53 signaling in autophagy-related cell death require further investigation. Surprisingly, BIX-01294 treatment did not change p53 protein level. It will be interesting to further investigate the mechanisms underlying the selective transcription of p53 induced by BIX-01294. BIX-01294 is an inhibitor for histone methyltransferase G9a which is a known methyltransferase for p53 [32]. It is possible that BIX-01294 regulates p53 methylation and other modifications without affecting its protein stability. Several reports have proved that the acetylation of the specific lysines on p53 selectively regulates its transcriptional activity [33–35]. So it will be highly interesting to study if BIX-01294 regulates p53 downstream gene transcription by modulating p53 methylation or other modifications on specific sites.

## Supporting Information

S1 File Table A, The list of chemical molecules used in the drug screen. Typical chemotherapy drugs and epigenetic inhibitors were used in the screen for SMYD2 novel functions. The known functional mechanisms of the drugs and a summary of screen results were also listed. **Table B, Different expressed genes in the BIX-01294-treated cell compared with control**. The RNA sequencing results were analyzed as described in Materials and Methods. The different expressed genes higher than 1.5 folds were listed. **Table C, Different expressed genes in the rapamycin-treated cell compared with control**. The different expressed genes higher than 1.5 folds were listed. **Table D, Different expressed genes in the 5-FU-treated cell compared with control**. The different expressed genes higher than 1.5 folds were listed. **Table E, Different expressed genes between SMYD2 knockdown cell and control**. HCT116 cell were transfected with siRNA targeting SMYD2 and sequenced for global mRNA expression. The different expressed genes higher than 1.5 folds were listed. **Table F, Different expressed genes of SMYD2 knockdown cell with or without BIX-01294 treatment**. SMYD2 was knocked down by siRNA and the different expressed genes higher than 1.5 folds after BIX-01294 treatment were listed. **Table G, Different expressed genes of SMYD2 knockdown cell with or without rapamycin treatment**. SMYD2 was knocked down by siRNA and the different expressed genes higher than 1.5 folds after rapamycin treatment were listed. **Table H, The list of primers for real time RT-PCR used in the study. Table I, The list of siRNA sequences targeting SMYD2 in the study**.
(XLSX)
